# ﻿Japanese *Tetramorium* queens: identification key and species diagnoses (Hymenoptera, Formicidae, Myrmicinae)

**DOI:** 10.3897/zookeys.1084.69767

**Published:** 2022-01-26

**Authors:** Seiki Yamane1, Shingo Hosoishi2, Fuminori Ito3

**Affiliations:** 1 Haruyama-chô, Kagoshima-shi 899–2704, Japan Unaffiliated Kagoshima Japan; 2 Institute of Tropical Agriculture, Kyushu University, Motooka 744, Nishi-ku, Fukuoka 819–0395, Japan Kyushu University Fukuoka Japan; 3 Faculty of Agriculture, Kagawa University, Ikenobe, Miki, Kagawa Pref. 761–0795, Japan Kagawa University Miki Japan

**Keywords:** Ant, caste difference, Japan, key to species, queen, species account, stat. rev., *Tetramorium*

## Abstract

In Japan, nine species have been known in the ant genus *Tetramorium*, of which five or more are considered tramps. A key to the queens of nine *Tetramorium* species found in Japan is presented. The tramp species *T.tonganum* Mayr, 1870 is excluded from the key because no queen was available for us, while *T.pacificum* Mayr, 1870 is included because it was once intercepted at a port in Japan and exotic queens were available. Diagnosis of the queen of each species is provided together with differences between the two female castes. *Tetramoriumtanakai* Bolton, 1977 is resurrected from synonymy with *T.kraepelini* Forel, 1905 based mainly on the queen characters.

## ﻿Introduction

*Tetramorium* Mayr, 1855 is a large ant genus containing slightly less than 600 species worldwide, with very few native species in the New World (AntWiki 2021). Most species are found in tropical and subtropical regions of Africa, Madagascar and Asia (e.g. Bolton 1977, 1980; Hita Garcia and Fisher 2012; Agavekar et al. 2017), though one group, the *T.caespitum* (Linnaeus, 1758) complex, has developed into more than ten species in northern Eurasia (e.g. Steiner et al. 2006). Some species are common, encountered during most field surveys on ant species diversity and some others are tramps spreading over non-native areas. In spite of their ubiquity, the nesting biology of *Tetramorium* species has not been studied well and many species are known only from the worker caste. In Japan nine species have been recorded, of which at least five are considered tramps or aliens (Terayama 2020; Yoshimura 2020). Although *Tetramoriumtanakai* Bolton, 1977 was synonymized with *T.kraepelini* Forel, 1905 by Japan Ant Database Group (2003), it is highly probable that it is a good species and it is resurrected from synonymy in the present paper. Species accounts for the known *Tetramorium* fauna of Japan are given by Terayama et al. (2014), based on the worker caste, but information about the queen and male is still very poor even for common species. The present paper is the first attempt to provide an identification key and morphological accounts for all the known Japanese species of the genus, except for *T.tonganum* Mayr, 1870. We include *T.pacificum* Mayr, 1870 that was once intercepted at a ferry port in Honshu. This paper is the second part of ‘the queens of the Japanese ants’ series; the first part dealt with the genus *Pheidole* (Yamane and Hosoishi 2020).

## ﻿Materials and methods

We tried to use queen specimens associated with workers, i.e. from colony series, for each species. Although we could examine only one queen for T.cf.kraepelini Forel, 1905, sampled by general collection, we identified it as T.cf.kraepelini, based on the key characters of the *T.kraepelini* species-complex, colouration and distribution information in Japan. As no queen of *T.tonganum*, either from Japan or outside Japan, was available, accordingly it has been omitted from the key. In the case of *T.pacificum*, not established yet in Japan, queens collected in Southeast Asia are used for the key and description. Specimens examined are deposited in the Entomological Laboratory, Kyushu University (KUEC: Fukuoka, Japan) and Seiki Yamane Collection (SKYC: Kitakyushu, Japan).

### ﻿Measurements

**TBL**: total body length roughly measured from anterior margin of head to tip of gaster; **HL**: maximum head length (in full-face view in a straight line from mid-point of anterior clypeal margin to mid-point of a transverse line spanning apices of projecting posteriormost portions of head); **HW**: maximum head width excluding eyes; **SL**: length of antennal scape; **EL**: maximum eye length (major diameter); **EW**: maximum eye width (minor diameter); **PtW**: maximum petiolar width; **PptW**: maximum postpetiole width; **CI**: cephalic index (HW divided by HL × 100); **SI**: scape index (SL divided by HW × 100); **ELI**: eye length index (EL divided by head length × 100). All measurements are expressed in mm.

### ﻿Identification and species accounts

#### List of the Japanese species of *Tetramorium*

*Tetramoriumbicarinatum* (Nylander, 1846) [*T.bicarinatum* group; introduced]

Tetramoriumcf.kraepelini Forel, 1905 [*T.scabrosum* group; ?native]

*Tetramoriumlanuginosum* Mayr, 1870 [*T.obesa* group; introduced]

*Tetramoriumnipponense* Wheeler, 1928 [*T.bicarinatum* group; native]

*Tetramoriumsimillimum* (F. Smith, 1851) *T.simillimum* group; introduced]

*Tetramoriumsmithi* Mayr, 1879 [*T.angulinode* group; introduced]

*Tetramoriumtanakai* Bolton, 1977, stat. rev. [*T.scabrosum* group; native]

*Tetramoriumtonganum* Mayr, 1870 [*T.tonganum* group: introduced]

*Tetramoriumtsushimae* Emery, 1925 [*T.caespitum* species group; native]

### ﻿Queen characters

The queens of the Japanese *Tetramorium* species, except for *T.tonganum* (queen not available) have the following character conditions in common: i) large eyes, ii) complete set of ocelli present, iii) full wings present (not confirmed in T.cf.kraepelini but distinct scars are seen at position of wing bases), iv) pronotum demarcated from mesonotum with distinct suture, v) mesonotum divided into mesoscutum and mesoscutellum, vi) notaulix absent, vii) mesopleuron divided into upper and lower portions, viii) distinctly defined metanotum and ix) metapleuron only weakly demarcated from lateral face of propodeum. In all Japanese species, the propodeal spine is moderate to well-developed and often stouter than in the worker. Ogata (1991) also provided a useful diagnosis for the queen caste of the Japanese species, but the species examined was not mentioned.

Amongst the Japanese myrmicine queens, *Tetramorium* queens are easily recognised by the following combination of the conditions: i) antennal club 3-segmented, ii) masticatory margin of mandible generally with seven teeth, iii) lateral portion of clypeus raised into sharp ridge that constitutes part of wall defining antennal insertion and iv) mid- and hind-tibiae each apically with simple spur. Important queen characters for identification of the Japanese *Tetramorium* species are listed below. Character conditions for the *T.tonganum* queen are cited from Bharti and Kumar (2012).

**Head shape.** Much broader than long: *T.tsushimae* Emery, 1925. Longer than broad or rarely almost as long as broad: other species.

**Mandibular sculpture.** Extensively smooth to superficially sculptured, shiny: *T.pacificum*, *T.smithi*. Almost entirely finely and densely striate, mat: other species.

**Antennal segments.** 11: *T.smithi*. 12: other species.

**Sculpture on antennal scrobe.** Of the same type as in surrounding areas: *T.tsushimae*. Simpler and weaker than in surrounding areas: other species.

**Anterior margin of clypeus.** Medially notched or impressed: *T.bicarinatum*, *T.nipponense*, *T.pacificum*. Entire: other species.

**Sculpture on mesonotum.** Principally puncto-reticulate, rugae if any wavy or indistinct: *T.lanuginosum*. Principally longitudinally striate/rugose: other species.

**Lower portion of mesopleuron.** Densely punctate or striate and mat: *T.simillimum* (punctate), *T.tsushimae* (striate). More or less shiny, with weak or superficial sculpture: other species.

**Petiolar length.** Very short; dorsal face seen from above 1/6 to 1/5 as long as broad: *T.tsushimae*. Much longer; dorsal face seen from above much more than 1/5 as long as broad: other species.

**Figure 1.**
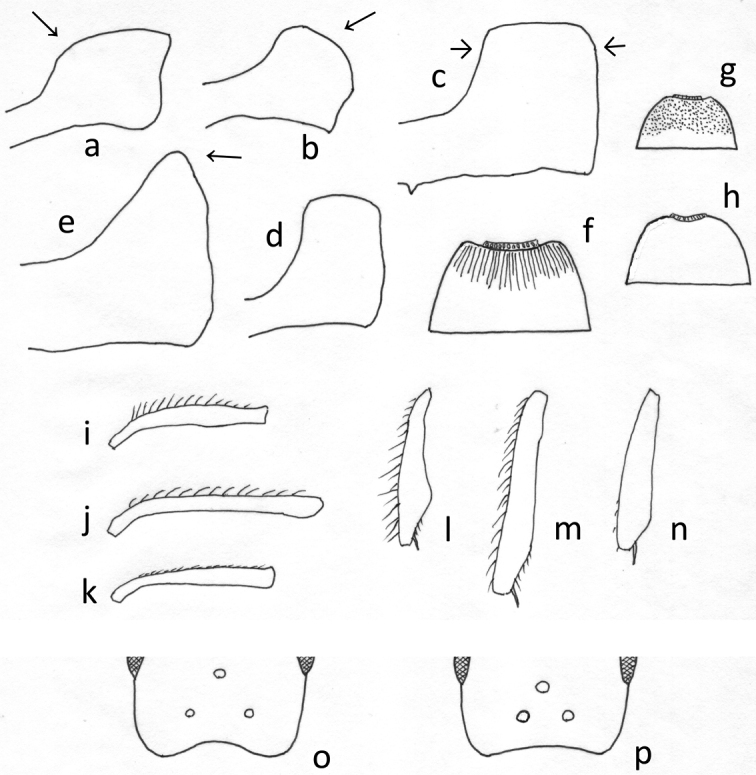
Some important characters used in the key to species **a–e** petiole in profile view **a***T.pacificum***b***T.lanuginosum***c***T.bicarinatum***d***T.smithi***e***T.tsushimae***f–h** anterior half of first gastral tergite **f***T.bicarinatum***g***T.simillimum***h***T.lanuginosum***i–k** antennal scape showing pilosity on its anterior margin **l–n** left hindtibia **i, l**T.cf.kraepelini**j, m***T.nipponense***k, n***T.simillimum***o, p** configuration of ocelli **o***T.tanakai***p**T.cf.kraepelini.

**Shape of petiolar node in profile view.** Anterior and dorsal faces confluent with continuous curve (Fig. 1a): *T.pacificum*. Dorsal face curving into posterior face without distinct angle between them (Fig. 1b): *T.lanuginosum*, *T.tonganum*. Anterior and posterior slopes more or less parallel and dorsum of node flat to weakly convex (Fig. 1c, d): other species except for *T.tsushimae* (Fig. 1e) (see above).

**Waist sculpture.** Dorsum of petiole or postpetiole, or both extensively smooth and shiny: T.cf.kraepelini, *T.smithi*, *T.tanakai*. Dorsum of petiole and postpetiole entirely densely sculptured: other species (in *T.tonganum* postpetiole dorsum almost smooth).

**Basal portion of gastral tergite 1** (abdominal tergite 4). Basal 1/6–1/3 of the tergite longitudinally striate (Fig. 1f): *T.bicarinatum*, *T.nipponense*, *T.pacificum*. Basal 1/3 micropunctate or microsculptured (sculpture can be superficial) and mat or weakly shiny (Fig. 1g): *T.simillimum*. Entire tergite essentially smooth and shiny (Fig. 1h): other species (in *T.tsushimae* this part is sometimes very superficially microsculptured, but this species is easily separable from others in other characters, see above).

**Body pilosity.** Bifid and trifid hairs present on dorsal body: *T.lanuginosum*. All body hairs simple: other species.

**Pilosity on antennal scape and mid- and hindtibiae.** Erect and suberect hairs abundant (in aged foundresses, hairs can be partly missing) (Fig. 1i, l): T.cf.kraepelini, *T.tanakai*. Principally decumbent or appressed, but variable (hairs on antennal scape can be near suberect, those on tibiae less frequently suberect) (Fig. 1j, m): *T.bicarinatum*, *T.nipponense*, *T.pacificum*, *T.tonganum*. All hairs decumbent or appressed (Fig. 1k, n): *T.lanuginosum*, *T.simillimum*, *T.smithi*, *T.tsushimae*.

For the wing venation in the Japanese species of *Tetramorium*, see Ogata (1991).

### ﻿Key to Japanese species (queens) excluding *T.tonganum*

**Table:** 

1	Basal 1/6–1/3 of gastral tergite 1 (abdominal tergite 4) with fine longitudinal striae (Fig. 1f) or micropunctures that are sometimes superficial; sculptured area mat or weakly shiny (Fig. 1g)	**2**
–	Entire gastral tergite 1 (abdominal tergite 4) essentially smooth; punctures very sparse and cuticular surface shiny (microsculpture if any confined to extreme basal area) (Fig. 1h)	**5**
2	Gastral tergite 1 basally with micropunctures and mat (sometimes weakly shiny) (Fig. 1g). Erect hairs on dorsum of head and mesosoma not tapering apicad, some with truncate apex. Anterior clypeal margin entire	***T.simillum***
–	Gastral tergite 1 longitudinally striate at base (Fig. 1f). Erect hairs on dorsum of head and mesosoma tapering apicad, some with pointed apex. Anterior clypeal margin medially with small notch or impression	**3**
3	Mandible extensively smooth. Petiolar node longer, with anterior slope gentle, curving into petiolar dorsum (Fig. 1a). Body dark reddish-brown (Fig. 2e). (Japanese specimen not available)	***T.pacificum***
–	Mandible entirely striate. Petiolar node shorter, with anterior slope almost vertical (Fig. 1c). Body yellowish-brown to brown often with darker gaster (Fig. 2a)	**4**
4	Petiole in profile with almost flat dorsal outline; its posterior slope generally straight (Fig. 1c). Propodeum with 2–3 strong transverse carinae between propodeal spines. Gaster blackish-brown	***T.bicarinatum***
–	Petiole in profile with shallowly convex dorsal outline; its posterior slope often shallowly concave. Transverse propodeal carinae absent or very weak (at most only one distinct carina present). Gaster yellowish-brown to brown	***T.nipponense***
5	Larger species with total body length 5.5–6 mm. Entire body dark brown to blackish (Fig. 3c). Petiolar node in profile very short (thin), without distinct dorsal face, with rounded apex (Fig. 1e)	***T.tsushimae***
–	Smaller species with total body length less than 4 mm. Body yellowish-brown to brown, sometimes with much darker gaster. Petiolar node in profile longer (thicker), generally with more or less distinct dorsal face (in *T.lanuginosum* dorsal face confluent to posterior declivity with smooth curve) (Fig. 1b, d)	**6**
6	Dorsum of head and mesosoma with many bifid and fewer trifid erect hairs. Petiolar node in profile view longer than high, of peculiar shape, namely dorsum smoothly continuous to posterior declivity. Dorsum of both petiole and postpetiole without smooth area	***T.lanuginosum***
–	Dorsum of head and mesosoma without bifid/trifid erect hairs. Petiolar node in profile shallowly convex dorsally or flat, as long as or shorter than high. Dorsum of petiole or postpetiole or both with smooth area	**7**
7	Antenna 11-segmented. Antennal scape and mid- and hind-tibiae without erect hairs; hairs appressed, decumbent or at most weakly suberect. Posterior declivity of propodeum without transverse carinae	***T.smithi***
–	Antenna 12-segmented. Antennal scape and mid- and hind-tibiae with many suberect to erect hairs (Fig. 1i, l). Posterior declivity of propodeum with some transverse carinae	**8**
8	Body entirely yellowish-brown (Fig. 2b). Posterior ocelli separated from each other by less than 2.5× ocellar diameter (Fig. 1p). Petiole in profile with anterodorsal corner rather sharply angled	**T.cf.kraepelini**
–	Body entirely dark reddish-brown (Fig. 3b). Posterior ocelli widely separated from each other by 4.5× ocellar diameter (Fig. 1o). Petiole in profile with anterodorsal corner more roundly angled	***T.tanakai***

### ﻿Species accounts

#### ﻿ Tetramorium bicarinatum

Taxon classificationAnimaliaHymenopteraFormicidae

(Nylander, 1846)

120C1EBC-B92C-57E6-B410-0CC223FFB1E1

**Figure 2.**
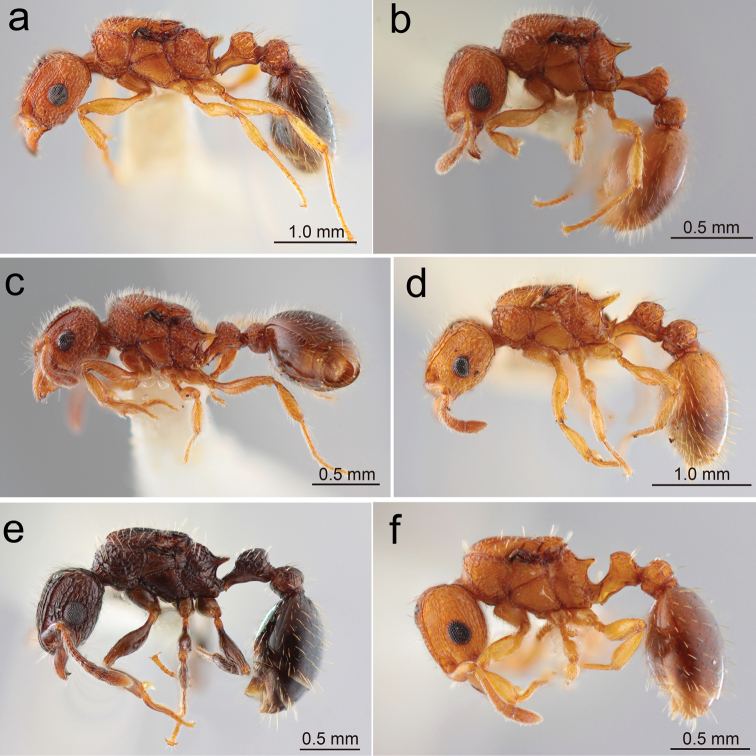
Japanese *Tetramorium* queens: habitus in profile view **a***T.bicarinatum* (Nagashima, Kagoshima-ken, Kyushu) **b**T.cf.kraepelini (Itoman, Okinawa-jima, Okinawa-ken) **c***T.lanuginosum* (Komi, Iriomote-jima, Okinawa-ken) **d***T.nipponense* (Umi-jinja, Shikano-shima, Fukuoka-shi) **e***T.pacificum* (Upper Thompson Nature Park, Singapore) **f***T.simillimum* (Yoron-jima, Amami Is., Kagoshima-ken). (Same specimens were used for ‘head in full-face view’ and ‘habitus in dorsal view’).

Figs 1c , 2a , 4a , 5a

##### Queen diagnosis.

Measurements (n = 5): TBL 3.9–4.9 (4.4), HL 1.04–1.09 (1.06), HW 0.9–0.95 (0.94), SL 0.64–0.70 (0.68), EL 0.28 (0.28), EW 0.23–0.26 (0.24), PtW 0.39–0.42 (0.41), PptW 0.50–0.52 (0.51), CI 85.7–91.6 (89.0), SI 67.1–75.5 (71.8), ELI 25.7–26.9 (26.5). Head and mesosoma yellowish-brown; gaster blackish-brown. Dorsum of head between frontal carinae with distinct rugae that are weakly waved. Clypeus with three longitudinal carinae; anterior margin with median notch (impression). Vertex, temple, gena, pronotum and nodes of petiole and postpetiole coarsely reticulate. Mandible densely striate. Antennal scape with long decumbent/suberect hairs. Mesonotum covered with rather regular longitudinal rugae. Posterior declivity of propodeum with 2–3 distinct transverse carinae between propodeal spines. Dorsum of petiole and postpetiole coarsely reticulate. Gastral tergite 1 with fine, dense longitudinal striae at base.

##### Caste difference.

Worker measurements (n = 5): TBL 2.5–3.3 (3.0), HL 0.83–0.94 (0.88), HW 0.7–0.8 (0.74), SL 0.56–0.61 (0.58), EL 0.18–0.21 (0.19), EW 0.16–0.18 (0.17), PtW 0.24–0.28 (0.26), PptW 0.31–0.37 (0.34), CI 81.9–85.3 (84.1), SI 76.3–81.4 (79.1), ELI 20.9–22.7 (21.8). Worker much smaller than the queen. Eye smaller; distance between mandibular base and anterior margin of eye longer than major diameter of eye; in the queen, the distance as long as or shorter than major diameter of eye. Mandible more weakly striate and more shiny than in the queen. Entire dorsum of mesosoma puncto-reticulate; in the queen mesonotum longitudinally rugose. Propodeal spine slender and always up-curved apically; in the queen it tends to be more strongly sclerotised, relatively shorter, with broader base than in the worker and apex not distinctly up-curved. Long hairs on antennal scape and mid- and hind-tibiae frequently near suberect; in the queen, these hairs less frequently near-suberect.

##### Specimens examined.

Kyushu mainland: 2q (dealate), Kyushu Univ. Hakozaki Campus, Fukuoka-shi, emerged from colony collected in ix. 2015 by M. Obika and kept in lab; 1q (dealate), Hongôkitakata, Miyazaki-shi, 21.vii.2020, Sk. Yamane & G. Mita; 1q (dealate), Naga-shima, Kagoshima-ken, 31.vii.1979, K. Ogata (Figs 2a, 4a, 5a); 4q (dealate), Kagoshima Univ. Kôrimoto Campus, Kagoshima-shi, 7.vi.2005, rotting log, Sk. Yamane leg. (JP05-SKY-101); 1q (winged), Kamitaniguchi, Kagoshima-shi, 16.ix.2008, attracted to light, Sk. Yamane leg. N. Ryukyus: 1q (dealate), Nakano-shima, Tokara Islands, 31.iii.1991, Y. Yamanouchi leg. C. Ryukyus: 2q (dealate), Takabaru, Yoro-shima, Amami Islands, 2.vii.2015, dead stem on tree, Sk. Yamane leg. (JP15-SKY-34); 1q (dealate), Shuri, Okinawa-jima, 17.vii.2020, in house, Y. Kusui leg.

##### Distribution

**in Japan.** Honshu (Pacific coast), Shikoku, Kyushu, throughout the Nansei Islands, Ogasawara Islands and Iwô Islands (Terayama et al. 2014).

##### Remarks.

*Tetramoriumbicarinatum* is an alien tramp species found in disturbed areas in warmer regions of the world except in Africa. It belongs to the *T.bicarinatum* species group (Bolton 1977) together with *T.nipponense* in Japan. In the queen this species is most similar to *T.nipponense*, which inhabits forests, preferring wetter conditions. The possession of 2–3 transverse carinae between the propodeal spines is an important characteristic in distinguishing the two species; in *T.nipponense* these carinae are lacking or much weaker (at most one distinct carina present). Furthermore, the propodeal spine is straight throughout its length in *T.bicarinatum*, while it tends to have a slightly up-curved apex in *T.nipponense*. New queens are attracted to light.

#### ﻿ Tetramorium cf. kraepelini

Taxon classificationAnimaliaHymenopteraFormicidae

Forel, 1905

915152C6-DE5D-593A-90C0-7F32D29D7CF6

Figs 1i, lp, 2b, 4b, 5b

##### Queen diagnosis.

Measurements (n = 1): TBL 2.7, HL 0.64, HW 0.58, SL 0.38, EL 0.21, EW 0.16, PtW 0.23, PptW 0.28, CI 90.6, SI 65.5, ELI 32.8. Body yellowish-brown. Frons between frontal carinae with weak longitudinal rugae. Clypeus with some weak longitudinal carinae; anterior margin of clypeus without median notch. Eye large, 2 times as long as distance between anterior margin of eye and mandibular base. Distance between posterior ocelli less than 2.5× ocellar diameter. Vertex, temple including posterolateral corner of head, dorsum of pronotum puncto-reticulate. Entire mesonotum with dense and longitudinal rugae; dorsum of propodeum longitudinally rugose, continuous to anterior margin of posterior declivity that is weakly transversely striate. Lateral face of pronotum and upper portion of mesopleuron distinctly striate; lower portion of mesopleuron only superficially sculptured and shiny. Petiole with strong carinae on lateral face, superficially sculptured and weakly shiny on dorsum; dorsum of postpetiole smooth and shiny. Gastral tergite 1 basally without carinae, smooth. Antennal scape and mid- and hind-tibiae with erect/suberect hairs.

##### Caste difference.

Worker measurements (n = 5): TBL 2.0–2.1 (2.1), HL 0.58–0.61 (0.60), HW 0.53–0.55 (0.54), SL 0.35–0.38 (0.36), EL 0.14–0.16 (0.15), EW 0.09–0.10 (0.10), PtW 0.17–0.18 (0.18), PptW 0.19–0.23 (0.22), CI 88.3–90.2 (90), SI 65.5–69.1 (67.3), ELI 23.7–26.2 (25.1). Worker smaller than the queen. In the worker, head more extensively reticulate, leaving area behind clypeus longitudinally rugose. Eye smaller than in the queen, as long as or only slightly longer than distance between anterior eye margin and mandibular base. Eye strongly converging anteriad; in the queen, eye broadly rounded anteriorly. Mesosomal dorsum entirely densely reticulate; in the queen, mesonotum with dense longitudinal striae. In the worker, both petiole and postpetiole dorsum smooth and shiny.

##### Specimens examined.

C. Ryukyus: 1q (dealate), Itoman, Okinawa-jima, 18.viii.1991, Y. Yamanouchi leg. (Figs 2b, 4b, 5b).

##### Distribution

**in Japan.** Kyushu and throughout the Nansei Islands (Terayama et al. 2014).

##### Remarks.

Tetramoriumcf.kraepelini belongs to the *T.scabrosum* group (sensu Bolton 1977). The so-called *T.kraepelini* can be a complex of sibling species. As only one queen was available for examination the variation in structure and sculpture is unknown. One queen from a colony collected from Central Thailand and tentatively identified as *T.kraepelini* is very similar to the queen examined above; however, the distance between the posterior ocelli is slightly longer than in the Japanese form.

#### ﻿ Tetramorium lanuginosum

Taxon classificationAnimaliaHymenopteraFormicidae

Mayr, 1870

BF8D9517-7432-5E7E-A163-249CD12113FF

Figs 1b, h , 2c , 4c , 5c

##### Queen diagnosis.

Measurements (n = 5): TBL 2.5–3 (2.7), HL 0.69–0.73 (0.71), HW 0.69–0.73 (0.7), SL 0.45–0.5 (0.46), EL 0.2–0.21 (0.2), EW 0.16–0.18 (0.17), PtW 0.27–0.28 (0.28), PptW 0.29–0.3 (0.29), CI 95.9–101.4 (98.6), SI 61.6–72.5 (65.9), ELI 27.4–30.4 (28.7). Body yellowish-brown, with gaster much darker. Head almost as long as broad. Frons medially with longitudinal rugae; other portions of head puncto-reticulate. Clypeus irregularly and superficially sculptured and shiny, with median carina. Eye large; distance between anterior eye margin and mandibular base much shorter than half eye length. Pronotum, mesonotum, lateral mesosoma, except for lower portion of mesopleuron and petiolar and postpetiole nodes puncto-reticulate except for lower portion of mesopleuron rather shiny (mesoscutum may have rather distinct longitudinal rugae). Peduncle of petiole smooth and shiny; dorsal face of petiole not defined, smoothly continuous to declivity; subpetiolar process almost missing. Gastral tergite 1 without longitudinal carinae at base. Vertex and mesosoma with many bifid and fewer trifid erect hairs; these hairs much fewer on gastral tergites.

##### Caste difference.

Worker measurements (n = 5): TBL 2.1–2.6 (2.4), HL 0.64–0.68 (0.65), HW 0.6–0.65 (0.62), SL 0.43 (0.43), EL 0.14–0.15 (0.15), EW 0.09–0.11 (0.1), PtW 0.18–0.23 (0.21), PptW 0.23–0.25 (0.24), CI 93.8–98.4 (95.4), SI 66.2–71.7 (69.2), ELI 22.1–23.4 (22.7). Worker consistently smaller than the queen. Head only slightly longer than broad in the worker. In the worker, dorsum of head rather extensively reticulate, with weak longitudinal rugae, and mesosomal dorsum densely reticulate, completely lacking rugae; in the queen, at least some rugae recognised on mesonotum. Lateral face of mesosoma entirely sculptured; in the queen, lower portion of mesopleuron more or less smooth and shiny. Eye smaller, only slightly longer than distance between anterior eye margin and mandibular base; in the queen, eye distinctly longer than the distance. Bifid hairs denser on gastral tergite 1 than in the queen.

##### Specimens examined.

C. Ryukyus: 11q (2 dealated, 9 winged), Takabaru, Yoro-jima, Amami Islands, 2.vii.2015, rotting branch on ground, Sk. Yamane leg. (JP15-SKY-27); 4q (3 winged, 1 dealate), Suehiro Park, Shuri, Okinawa-jima, Okinawa Is., emerged from colony (FI19–25) collected on 11.iii.2019 from soil and reared in lab. S. Ryukyus: 5q (winged), Ishigaki-jima, Yaeyama Is., emerged from colony (FI19–15) collected on 7.ii.2019 from under stone in forest and reared in lab; 1q (dealate), Komi, Iriomote-jima, Yaeyama Is., 16.v.1979, K. Ogata leg. (Figs 2c, 4c, 5c); 7q (4 winged, 3 dealate), Inbi-dake, Yonaguni-jima, Yaeyama Is., emerged from colony (FI15–96) collected on 7.xi.2015 from decayed wood in forest and reared in lab.

##### Distribution

**in Japan.** Nansei Islands. The northern limit lies in Kuchinoerabu-jima and Tanega-shima of the Ôsumi Islands (Yamane and Fukumoto 2017).

##### Remarks.

*Tetramoriumlanuginosum* belongs to the *T.obesa* species group of the former ‘*Triglyphothrix* Forel, 1890’ (Bolton 1976). This group is characterised by the mixture of simple and bifid hairs on the body, with fewer or no trifid hairs. *Tetramoriumlanuginosum* queens have many bifid hairs on the head and mesosoma (very few on gastral tergite 1), but trifid hairs are absent on the gaster and fewer on head and mesosoma. This species is easily distinguished in both the worker and queen by the presence of bifid and trifid hairs on the body, erect hairs on the antennal scape and mid- and hind-tibiae and the petiole without distinction of the dorsum from posterior declivity.

#### ﻿ Tetramorium nipponense

Taxon classificationAnimaliaHymenopteraFormicidae

Wheeler, 1928

A6620980-367A-52A3-8F6A-E6E9EDC8DF47

Figs 1j, m , 2d , 4d , 5d

##### Queen diagnosis.

Measurements (n = 5): TBL 3.5–4 (3.8), HL 0.83–0.86 (0.85), HW 0.69–0.76 (0.72), SL 0.54–0.58 (0.56), EL 0.23–0.25 (0.24), EW 0.18–0.2 (0.19), PtW 0.28–0.33 (0.3), PptW 0.36–0.41 (0.39), CI 82.4–88.4 (85.1), SI 74.7–80 (77.6), ELI 27.1–29.8 (28.1). Body yellowish-brown, with gaster slightly darker; coxae, femora and tibiae of all legs creamy yellow. Head distinctly longer than broad. Head reticulate, except for frons between clypeus and ocellar area with a few longitudinal carinae; clypeus superficially sculptured and shiny, with three longitudinal carinae. Mandible densely striate. Pronotal dorsum coarsely reticulate; mesonotum longitudinally striate/rugose; propodeum irregularly sculptured and shiny; transverse carinae between propodeal spines absent or weak (at most only one distinct carina present). Lateral face of mesosoma coarsely rugose except for lower portion of mesopleuron weakly sculptured; propodeal spine slender, often weakly up-curved apically. Nodes of petiole and postpetiole entirely puncto-reticulate. Gastral tergite 1 with longitudinal basal striae. Antennal scape and mid- and hind-tibiae with many decumbent to near suberect hairs.

##### Caste difference.

Worker measurements (n = 5): TBL 2.8–3.0 (2.9), HL 0.74–0.79 (0.77), HW 0.63–0.7 (0.66), SL 0.49–0.55 (0.53), EL 0.16–0.18 (0.17), EW 0.12–0.14 (0.13), PtW 0.23–0.26 (0.25), PptW 0.28–0.33 (0.30), CI 84.0–88.6 (86.4), SI 75.4–85.7 (80.2), ELI 24.6–27.3 (25.9). Worker very similar to the queen in coloration, structure and sculpture, with the following differences. Body smaller. Eye smaller, distance between anterior margin of eye and mandibular base slightly longer than major diameter of eye; in the queen, the distance slightly shorter than major diameter of eye. Dorsum of mesosoma entirely coarsely reticulate; in the queen, mesonotum with longitudinal rugae/striae that are dense and often irregular. Lower portion of mesopleuron sculptured and slightly mat; in the queen, lower portion with much weaker sculpture and shiny. Propodeal spine up-curved in apical 1/3; in the queen, the spine tending to be straighter throughout. Petiole tends to be longer than in the queen. Long hairs on antennal scape and mid- and hind-tibiae frequently near-suberect; in the queen, these hairs less frequently near suberect.

##### Specimens examined.

Kyushu: 1q (dealate), Umi-jinja, Shikano-shima, Fukuoka-shi, 15.ix.1980, K. Ogata leg. (Figs 2d, 4d, 5d); 6q (winged), Yoshino-chô, Kagoshima-shi, 7.ix.2016, attracted to light, T. Tsukada leg.; 3q (dealate), Kenkôno-mori, Inusako-chô, Kagoshima-shi, 20.iv.2019, dry dead twig on ground, Sk. Yamane leg. (JP19-SKY-022); 3q (dealate), near Hetsuka, Minamiôsumi-chô, Kagoshima-ken, 23.vii.2020, nest under moss, Sk. Yamane leg. (JP20-SKY-069). N. Ryukyus: 1q (dealate), Tashiro-Yumugi, Kuchinoerabu-jima, Ôsumi Islands, 26.vii.2016, nest in dead sasa bamboo, Sk. Yamane leg. (JP16-SKY-70); 2q (dealate), Maeda, Kuchinoerabu-jima, in dead twig on ground, Sk. Yamane leg. (JP16-SKY-120). C. Ryukyus: 1q (dealate), Nagakumo, Tatsugô-chô, Amami-ôshima, Amami Islands, 22.xii.2015, in decayed wood, Sk. Yamane leg. (JP15-SKY-72); Takahachi-yama, Amami-ôshima, 5.iii.2017, in decayed wood, Sk. Yamane leg. (JP17-SKY-20); 7q (winged), Chinase, Naze, Amami-ôshima, 28.viii.2019, attracted to light, K. Kanai leg.; 3q (2 winged, 1 dealate), Yonaha, Okinawa-jima, 5.vii–5.viii.2001, Malaise trap, T. Muroi & C. Nakamura.

##### Distribution

**in Japan.** Honshu (southern coast), Shikoku, Kyushu, throughout Nansei Islands, Senkaku Islands.

##### Remarks.

*Tetramoriumnipponense* belongs to the *T.bicarinatum* species group (Bolton 1977) together with *T.bicarinatum* in Japan. The gaster is generally much paler in colour than in the latter. The shape of the petiole that is frequently used to distinguish between these species in the worker caste is not very useful in the queen, though in *T.nipponense* the petiole tends to be longer and have a weakly convex dorsal face (for further discussion, see *Remarks* under *T.bicarinatum*). Queens of this species are frequently attracted to light and also caught with Malaise traps.

#### ﻿ Tetramorium pacificum

Taxon classificationAnimaliaHymenopteraFormicidae

Mayr, 1870

8C3ADC28-FE60-52A4-B290-2C7C7894E79D

Figs 1a , 2e , 4e , 5e

##### Queen diagnosis.

Measurements (n = 3; material from Southeast Asia): TBL 3.5–3.8 (3.6), HL 0.81–0.86 (0.84), HW 0.70–0.74 (0.73), SL 0.53–0.55 (0.54), EL 0.21–0.23 (0.22), EW 0.16–0.18 (0.17), PtW 0.28–0.30 (0.29), PptW 0.35–0.37 (0.36), CI 86.0–88.1 (86.8), SI 71.6–75.7 (73.9), ELI 24.4–28.4 (26.7). Body brown to dark reddish or blackish-brown. Head in full-face view longer than broad, with almost parallel lateral margins. Frons sparsely longitudinally rugose with interspaces superficially sculptured and often shiny; other portions of head puncto-reticulate; antennal scrobe densely but weakly sculptured and rather shiny. Clypeus with three parallel longitudinal carinae; its anterior margin medially impressed but more weakly than in *T.bicarinatum* and *T.nipponense*. Mandible very superficially sculptured or almost smooth and shiny. Pronotal dorsum puncto-reticulate; mesonotum with parallel longitudinal rugae that are often wavy or irregular; dorsum and lateral face of propodeum puncto-reticulate or irregularly coarsely sculptured; remaining portions of mesosoma mainly striate to rugose but lower portion of mesopleuron generally smoother and shiny. Petiole in profile long; its node longer than high, with anterior slope gentle and not clearly separable from dorsum and steep posterior slope; petiole and postpetiole both dorsally and laterally distinctly sculptured and mat. Basal 1/3 or more of gastral tergite 1 covered with distinct longitudinal striae. Antennal scape and mid- and hind-tibiae without erect hairs.

##### Caste difference.

Worker measurements (n = 7; 3 specimens intercepted at Kure Port, Japan and 4 from Southeast Asia): TBL 2.9–3.6 (3.3), HL 0.83–0.95 (0.88), HW 0.70–0.81 (0.75), SL 0.53–0.64 (0.60), EL 0.16–0.20 (0.19), EW 0.13–0.14 (0.13), PtW 0.24–0.29 (0.27), PptW 0.29–0.37 (0.33), CI 83.3–87.9 (85.5), SI 75.7–82.9 (80.0), ELI 19.3–22.7 (21.3). Worker very similar to the queen, but with the following characteristics that are different from those of the latter. Body slightly, but constantly larger than in the queen in terms of HL, HL and SL, but eye size smaller than in the latter with ELI 21.3 (worker) vs. 26.7 (queen). Distance between anterior eye margin and mandibular base distinctly longer than major diameter of eye; in the queen, the distance as long as major diameter of eye. Entire dorsum of mesosoma coarsely puncto-reticulate; in the queen mesonotum longitudinally rugose. Mesopleuron entirely with dense minute punctures; in the queen, lower portion of mesopleuron rather smooth and sculpture in upper portion weak.

##### Specimens examined.

No Japanese specimen available. A dealate queen collected in Upper Thompson Nature Park, Singapore on 9.xii.2017 by Sk. Yamane was used for photos (Figs 2e, 4e, 5e).

##### Distribution

**in Japan.** Not established in Japan.

##### Remarks.

*Tetramoriumpacificum* belongs to the *T.bicarinatum* group, in which the anterior margin of the clypeus is notched or impressed at the middle (Bolton 1977). Amongst the species found in Japan, this species is easily distinguished from the others by having a near-smooth mandible and the anterior and dorsal faces of the petiole confluent with a smooth curve. Workers were found in a container at Kure Port, Hyogo-ken, Honshu, transported by a ferry from Zhongshan, China via Hiroshima Port. Up to now, any established population has not yet been confirmed in Japan.

#### ﻿ Tetramorium simillimum

Taxon classificationAnimaliaHymenopteraFormicidae

(F. Smith, 1851)

02A8C4F4-46F3-56C7-82FD-AFE7A1682BA8

Figs 1g, k, n , 2f , 4f , 5f

##### Queen diagnosis.

Measurements (n = 4): TBL 2.3–2.6 (2.5), HL 0.60–0.63 (0.62), HW 0.53–0.58 (0.56), SL 0.39–0.43 (0.41), EL 0.17–0.18 (0.18), EW 0.13 (0.13), PtW 0.20–0.23 (0.22), PptW 0.25–0.28 (0.27). Body yellowish-brown, with darker gaster and light brown to yellow legs. Dorsum of head with dense and longitudinal striae that are regular and parallel; interspaces microsculptured and mat. Clypeus with strong median carina; other longitudinal carinae weak, indistinct; anterior margin entire. Distance between anterior eye margin and mandibular base distinctly shorter than major diameter of eye. Dorsum of mesosoma entirely sculptured; pronotum reticulate, with anterolateral corner angulate; entire mesonotum densely with longitudinal striae; in profile lateral face of mesosoma entirely sculptured; upper portion of mesopleuron rugose and lower portion densely punctate (sometimes punctation weak). Propodeum with longitudinally puncto-striate dorsum and transversely puncto-striate declivity, entirely mat, without transverse carinae between propodeal spines; propodeal spine short, apically blunt, only slightly longer than metapleural lobe. Petiolar node anteriorly sharply truncate; petiole and postpetiole entirely sculptured; ventre of petiolar peduncle superficially sculptured and weakly shiny; nodes of both petiole and postpetiole coarsely punctured. Gastral tergite 1 in basal 1/3 densely and minutely punctate or coriaceous, mat or weakly shiny. Antennal scape and mid- and hind-tibiae without erect hairs. Erect body hairs not tapering apicad, apically often truncate or blunt.

##### Caste difference.

Worker measurements (n = 5): TBL 1.8–2.1 (1.9), HL 0.53–0.59 (0.56), HW 0.45–0.53 (0.49), SL 0.33–0.39 (0.36), EL 0.11–0.13 (0.12), EW 0.08–0.09 (0.08), PtW 0.16–0.18 (0.17), PptW 0.19–0.23 (0.21), CI 83.3–89.8 (87.4), SI 71.7–77.6 (74.6), ELI 20.0–22.6 (21.1). Worker very similar to the queen, but with the following differences. Body smaller than in the queen. Distance between anterior eye margin and mandibular base as long as or slightly longer than major diameter of eye; in the queen, the distance much shorter than major eye diameter. Mesonotum coarsely rugoso-reticulate, rugae wavy and irregular; in the queen striae fine and regular. Gastral tergite 1 entirely smooth and shiny; in the queen basal area of the tergite micropunctured and mat (sometimes microsculpture very faint and cuticle weakly shiny). Apical truncation of erect hairs on head and mesosoma more distinct; in the queen, erect hairs often not typically truncate.

##### Specimens examined.

C. Ryukyus: 1q, (dealate), Nagahama-chô, Naze, Amami-ôshima, 17.vi.2017, in dead twig on ground, F. Ito leg.; 1q (dealate), same loc., date and nesting site, F. Ito (FI17–102); 2q (dealate), Yoron-jima, 28.v–2.vi.1999 (Figs 2f, 4f, 5f).

##### Distribution

**in Japan.** Nansei Islands, Ogasawara Islands, Volcano Island. Northern limit lies in Kodakara-jima of the Tokara Islands (Yamane and Fukumoto 2017).

##### Remarks.

*Tetramoriumsimillimum* belongs to the *T.simillimum* species group (Bolton 1977). In the queen, it is easily distinguished from other Japanese congeners by the following character conditions: i) dorsum of head with regular longitudinal striae, with interspaces densely sculptured and mat; ii) erect body hairs not sharply pointed apically; iii) petiole anteriorly truncate, with dorsal face clearly separated from anterior slope with sharp angle; iv) basal 1/3 of gastral tergite 1 micropunctate or coriaceous and mat (sometimes weakly shiny). This species is a famous tramp of African origin (Bolton 1977; Yoshimura 2020).

#### ﻿ Tetramorium smithi

Taxon classificationAnimaliaHymenopteraFormicidae

Mayr, 1879

1D9040B9-36E0-55CC-BAD7-9255CAC31301

**Figure 3.**
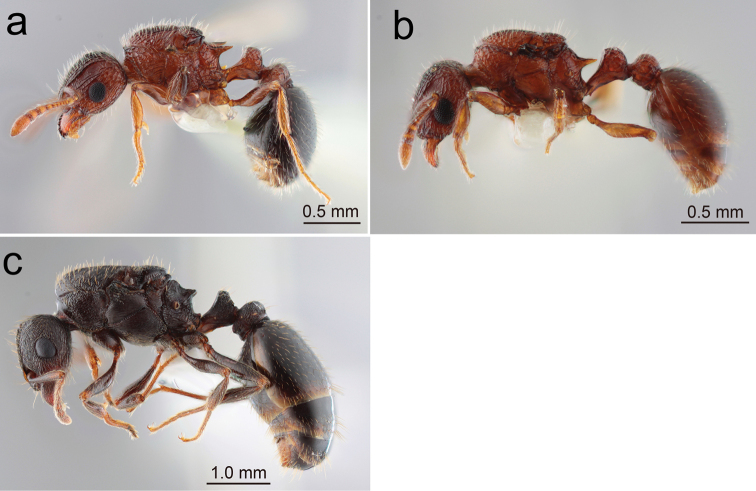
Japanese *Tetramorium* queens: habitus in profile **a***T.smithi* (Hirarahigashi, Miyako-jima, Okinawa-ken) **b***T.tanakai* (Mandabaru, Yonaguni-jima, Okinawa-ken) **c***T.tsushimae* (Nokono-shima, Fukuoka-shi). (Same specimens were used for ‘head in full-face view’ and ‘habitus in dorsal view’).

**Figure 4.**
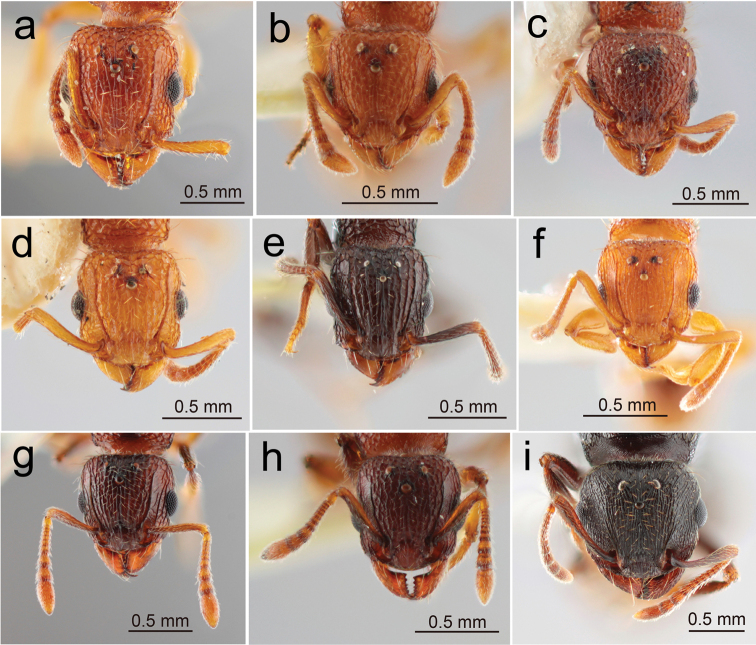
Japanese *Tetramorium* queens: head in full-face view **a***T.bicarinatum***b**T.cf.kraepelini**c***T.lanuginosum***d***T.nipponense***e***T.pacificum***f***T.simillimum***g***T.smithi***h***T.tanakai***i***T.tsushimae*.

Figs 1d , 3a , 4g , 6a

##### Queen diagnosis.

Measurements (n = 2): TBL 3.4/3.1, HL 0.73/0.74, HW 0.70/0.71, SL 0.43/0.43, EL 0.21/0.19, EW 0.17/0.17, PtW o.29/0.27, PpW 0.42/0.38, CI 95.9/95.9, SI 61.4/60.6, ELI 28.8/25.7. Head, mesosoma and waist brown to reddish-brown, with dorsum of head, antenna and legs dark brown; gaster black. Dorsum of head longitudinally rugose; other portions of head puncto-reticulate. Frontal carina strong, defining dorsal margin of antennal scrobe; antennal scrobe superficially sculptured and shiny. Clypeus with three longitudinal carinae, with anterior margin entire. Mandible superficially sculptured and shiny. Antenna with 11 segments. With mesosoma in dorsal view, pronotum sparsely transversely rugose; mesonotum and propodeal dorsum densely with longitudinal parallel rugae; propodeal declivity partly smooth, shiny; transverse carinae absent between propodeal spines; in profile view, pronotum, metapleuron and lateral face of propodeum rugose or irregularly sculptured; mesopleuron, especially on lower portion, with weaker sculpture and somewhat shiny. Petiole and postpetiole with smooth portions on their dorsum; petiolar peduncle and sternite smooth and shiny; lateral face of petiolar node and lateral and ventral faces of postpetiole sculptured; petiole ventrally with sharp longitudinal ridge throughout; postpetiole more than 1.4 times as broad as petiole in dorsal view. Gastral tergite 1 entirely smooth and shiny. Hairs on antennal scape and mid- and hind-tibiae decumbent to appressed; those on tibiae slightly approaching suberect.

##### Caste difference.

Worker measurements (n = 5): TBL 2.1–2.3 (2.2), HL 0.64–0.68 (0.65), HW 0.56–0.60 (0.59), SL 0.38–0.41 (0.40), EL 0.15–0.17 (0.16), EW 0.10–0.12 (0.11), PtW 0.19–0.23 (0.21), PptW 0.28–0.30 (0.29), CI 87.5–93.8 (90.5), SI 64.4–69.5 (67.4), ELI 23.4–25.0 (24.0). Worker much smaller than the queen. Eye smaller, distance between anterior eye margin and mandibular base as long as or slightly longer than major diameter of eye; in the queen the distance much shorter than major diameter of eye. Eye anteriorly distinctly tapered; in the queen eye anteriorly broadly rounded. Pronotal dorsum rugose except for small area around anterolateral corner reticulate; in the queen pronotal dorsum coarsely reticulate. Mesonotum longitudinally rugose in both castes. Mesopleuron entirely coarsely sculptured; in the queen, sculpture weaker, especially in lower portion.

##### Specimens examined.

S. Ryukyus: 1q (winged), Miyako-jima, 30.x.2019, nest in soil, on forest pass, F. Ito leg. (FI19–209); 1q (winged), Hirarahigashi, Miyako-jima, emerged on 28.vii.2020 in colony (FI19–203) collected on 29.x.2019 and kept in lab., F. Ito leg. (Figs 3a, 4g, 6a)

##### Distribution in Japan.

Nansei Islands. The northern limit lies in Okinawa-jima (Central Ryukyu Islands) (Terayama et al. 2014).

##### Remarks.

*Tetramoriumsmithi* belongs to the *T.angulinode* species group, in which the antenna has only eleven segments in the female castes (Bolton 1977). This species is easily separated from other Japanese congeners by the reduced antennal segments (11), shiny mandible (shared by *T.pacificum*) and black gaster (shared by *T.tsushimae*). This is the only Japanese *Tetramorium* species with the mesonotum entirely regularly rugose in both female castes.

#### ﻿ Tetramorium tanakai

Taxon classificationAnimaliaHymenopteraFormicidae

Bolton, 1977 stat. rev.

2F920BAF-40E7-59C0-9497-A94FC6D967AF

Figs 1o , 3b , 4h , 6b

Tetramorium tanakai Bolton, 1977: 119–120, Mt. Omoto, Ishigaki I.; Onoyama 1980: 198; Ogata 1991: 102; Bolton 1995: 415.
Tetramorium kraepelini : Japanese Ant Database Group 2003: 136; Terayama 2020: 120.

##### Queen diagnosis.

Measurements (n = 1): TBL 2.63, HL 0.66, HW 0.61, SL 0.40, EL 0.19, EW 0.16, PtW 0.30, CI 92.4, SI 65.6, ELI 28.8. Body brown to dark reddish-brown, with gaster very dark. Head reticulate, with frons between clypeus and ocellar region longitudinally rugose. Clypeus with three longitudinal carinae; its anterior margin entire. Posterior ocelli widely separated from each other; distance between them as long as 4.5× ocellar diameter. Dorsum of pronotum and propodeum coarsely reticulate; mesonotum longitudinally rugose (rugae on mesoscutellum irregular); lateral face of pronotum, upper portion of mesopleuron, metapleuron and lateral face of propodeum largely striate/rugose; lower portion and part of upper portion of mesopleuron smooth to very weakly sculptured and shiny; propodeal declivity irregularly sculptured; no transverse carinae between propodeal spines. Petiolar node entirely reticulate; sternite and peduncle microsculptured and mat; postpetiole with smooth area on its dorsum; its lateral face and sternite irregularly sculptured. Gastral tergite entirely smooth and shiny. Antennal scape and mid- and hind-tibiae with many erect hairs.

##### Caste difference.

Worker measurements (n = 5): TBL 2.0–2.4 (2.3), HL 0.59–0.63 (0.61), HW 0.53–0.58 (0.56), SL 0.36–0.40 (0.38), EL 0.13–0.16 (0.14), EW 0.08–0.10 (0.09), PtW 0.17–0.23 (0.20), PptW 0.20–0.25 (0.23), CI 86.4–93.2 (91.2), SI 65.5–70.6 (68.6), ELI 22.0–25.4 (23.1). Worker very similar to the queen in structure and sculpture, but differing in the following aspects. Eye smaller, distance between anterior eye margin and mandibular base as long as or slightly longer than major diameter of eye; in the queen the distance much shorter than major diameter of eye. Eye distinctly tapered anteriad; in the queen, eye anteriorly broadly rounded. Mesosomal dorsum entirely coarsely reticulate; mesonotum longitudinally rugose in the queen. Mesopleuron entirely sculptured; sculpture on mesopleuron much weaker especially in lower portion that is extensively smooth and shiny in the queen.

##### Specimens examined.

S. Ryukyus: 1q (dealate), Mandabaru, Yonaguni-jima, emerged in v.2020 in colony (FI19–108) collected on 14.iii.2019 by R. Hosokawa and kept in lab (Figs 3b, 4h, 6h). 1q (dealate), Yonaha-dake, Yonaguni-jima, 12.iii.2020, F. Ito (FI20–45).

##### Distribution in Japan.

Yaeyama Islands of the Ryukyu Islands (Ishigaki-jima and Yonaguni-jima).

##### Remarks.

*Tetramoriumtanakai* was originally described based on the worker and queen castes collected on Ishigaki-jima, Yaeyama Islands, Japan (Bolton 1977; no queen description provided). The worker is very similar to the Southeast Asian *T.kraepelini*, but is distinguished from the latter by the bicoloured body (dark brown head and gaster contrasted with lighter mesosoma, waist and legs) and the petiolar dorsum longer than the height of the tergal portion of the petiole (Bolton 1977). The workers sampled from Yonaguni-jima and examined in this study had a nearly entirely dark brown body with yellowish antennae and legs and are clearly different from extensively yellowish-brown workers of *T.kraepelini* and the Japanese T.cf.kraepelini. The dorsal length of the petiole is variable, generally as long as or slightly longer than the height of the tergal portion. The present study shows that the queen collected from Yonaguni-jima is also similar to that of T.cf.kraepelini in structure and sculpture, but clearly different from the latter in the widely separated posterior ocelli and coarsely reticulate dorsal propodeum as well as much darker body. We also examined queens and workers from two colonies collected in Shuisheliao, Taiwan. These specimens have a more typical bicolorous body and longer petiolar dorsum as in the original description of *T.tanakai*. The queens have widely separate posterior ocelli as in the queen from Yonaguni-jima. We consider the populations of Ishigaki-jima, Yonaguni-jima and Taiwan all belonging to the same species, *T.tanakai*. As species delimitation among the *T.kraepelini*-complex is very confusing, we need to have more colony series from various localities.

**Figure 5.**
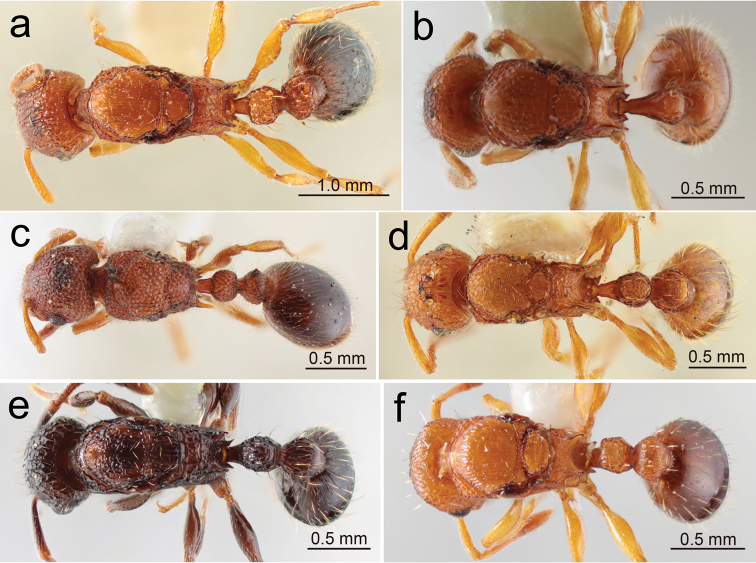
Japanese *Tetramorium* queens: habitus in dorsal view **a***T.bicarinatum***b**T.cf.kraepelini**c***T.lanuginosum***d***T.nipponense***e***T.pacificum***f***T.simillimum*.

**Figure 6.**
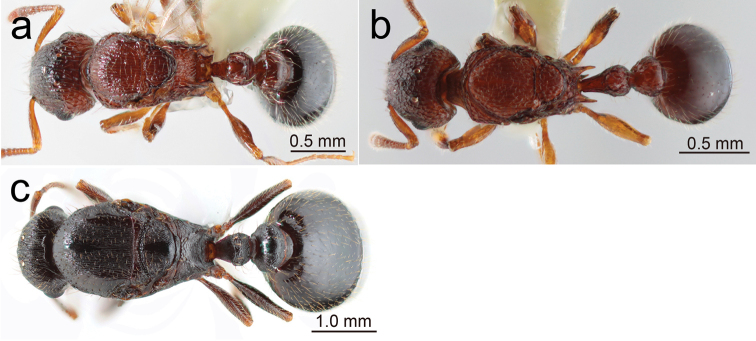
Japanese *Tetramorium* queens: habitus in dorsal view **a***T.smithi***b***T.tanakai***c***T.tsushimae*.

#### ﻿ Tetramorium tonganum

Taxon classificationAnimaliaHymenopteraFormicidae

Mayr, 1870

8117D691-E039-5026-908E-8AA8B369AA57

##### Remarks.

This species is a well-known tramp distributed from New Caledonia through the Indo-Australian region to China and Japan and has been introduced to remote islands in the Pacific and Atlantic Oceans (AntWiki 2021). In Japan, it has been recorded from the Ogasawara Islands (Bolton 1977; Terayama et al. 2014). We have no queen specimen of this species from Japan or from any other country. Santschi (1924) described the queen caste, based on a single specimen collected in Samoa. However, this specimen has only one ocellus and, according to his drawing, the eye is located too posteriorly on the head for a queen of *Tetramorium*. Bharti and Kumar (2012) described the queen caste of this species, based on material collected from Himachal Pradesh, India. According to their description (text and pictures), the queen of this species is recognised by the following combination of characteristics: i) anterior margin of clypeus entire, ii) no branched hairs present on body, iii) petiole in profile view with rounded anterodorsal and posterodorsal corners, iv) mid- and hind-tibiae without erect hairs, v) smooth lower portion of mesopleuron and vi) entirely smooth first gastral tergite.

#### ﻿ Tetramorium tsushimae

Taxon classificationAnimaliaHymenopteraFormicidae

Emery, 1925

6AF451BA-C780-505F-8B9F-F7A090C80095

Figs 1e , 3c , 4i , 6c

##### Queen diagnosis.

Measurements (n = 5): TBL 5.9–7.0 (6.4), HL 1.14–1.18 (1.17), HW 1.24–1.31 (1.27), SL 0.79–0.90 (0.84), EL 0.33–0.35 (0.34), EW 0.25–0.28 (0.26), PtW 0.45–0.50 (0.47), PptW 0.68–0.77 (0.74), CI 108.5–110.2 (109.1), SI 62.3–70.3 (66.2), ELI 28.0–29.7 (28.9). Body dark reddish-brown to blackish-brown. Head distinctly broader than long (CI ca. 109); entire head densely and regularly striate, with reticulate area restricted. Frontal carina weak and antennal scrobe barely recognisable. Clypeus with around ten longitudinal carinae; anterior margin entire. Mesosoma extensively striate to rugose, with smooth areas in anteromedian portion and area along parapsidal line of mesoscutum and longitudinal median zone of mesoscutellum; sculpture on pronotum, metapleuron and lateral face of propodeum coarser than in other areas; propodeum with striation longitudinal and irregular on dorsum and dense and transverse on declivity; interspaces minutely punctate. Metapleural lobe low, generally with round apex. Petiole in dorsal view dorsum very short (often indistinct), 1/6 to 1/5 as long as broad; postpetiole very broad, 1.57× as broad as petiole. Gastral tergite 1 entirely smooth, but sometimes with very superficial sculpture near base. All erect hairs simple and more or less tapering apicad. Antennal scape and mid- and hind-tibia without erect hairs.

##### Caste difference.

Worker measurements (n = 5): TBL 2.48–2.88 (2.71), HL 0.70–0.88 (0.77), HW 0.63–0.84 (0.71), SL 0.53–0.63 (0.57), EL 0.13–0.17 (0.15), EW 0.10–0.12 (0.11), PtW 0.18–0.28 (0.23), PptW 0.25–0.35 (0.29), CI 88.7–95.5 (92.1), SI 75.0–87.3 (81.1), ELI 18.1–18.3 (19.2). Worker very similar to the queen in colouration, structure, sculpture and pilosity, but differing in the following aspects: body much smaller; head longer than broad (CI ca. 92 vs. ca. 109 in the queen.); striation on mesonotum sparser and more irregular than in the queen; petiole more globular than in the queen, with rather distinct dorsum, which is in dorsal view only slightly broader than long; both petiole and postpetiole dorsally with smooth areas; in the queen almost entirely sculptured.

##### Specimens examined.

Honshu: 1q (dealate), Mihagi-dai, Hagi-shi, Yamaguchi-ken, 12.x.2013, nest under stone, Sk. Yamane leg. (JP13-SKY-27); 1q (dealate), Niino-hama, Heki, Nagato-shi, Yamaguchi-ken, 12.x.2013, nest under stone, Sk. Yamane leg. (JP13-SKY-28). Kyushu: 3q (dealate), Nokonoshima, Fukuoka-shi, Fukuoka-ken, 3.viii.2020, under stone, S. Hosoishi (SH20-Jpn-01) (Figs 3c, 4i, 6c); 2q (winged), Haruyama, Matsumoto-chô, Kagoshima-ken, 15.vi.2000, nest in soil, Sk. Yamane leg. (KG00-SKY-02).

##### Distribution in Japan.

Hokkaido, Honshu, Shikoku, Kyushu, Ôsumi Islands, Tokara Islands. The southern limit lies in Suwanose-jima of the Tokara Islands (Yamane and Fukumoto 2017).

##### Remarks.

*Tetramoriumtsushimae* belongs to the *T.caespitum* species group. In the queen caste this species is easily distinguished from other Japanese congeners by the large and blackish-brown body, broad head, ill-defined antennal scrobe, presence of smooth areas on the mesonotum and very short petiole.

## ﻿Discussion

This paper is the first attempt to prepare a key to Japanese *Tetramorium* species, based on the queen and to give morphological diagnoses for them. Material is still very poor; only one or two queens were available for two species, i.e. T.cf.kraepelini and *T.tanakai*. This shortfall means that the results of this study are tentative, needing further material from colony series.

Nevertheless, we have a strong impression that *Tetramorium* queens can be sorted into species as for workers. We found some useful characters for identifying queens. For example, in the worker caste, *T.bicarinatum* is distinguished from *T.nipponense* mainly based on the relative length of erect hairs along the frontal carina (compared with maximum eye length) and the shape of the petiole. However, these characters are less reliable for the queen. The presence/absence of distinct transverse carinae between propodeal spines is more decisive, and, finally, this character has proved to be also useful for the worker caste (this has not been mentioned by previous authors including Bolton 1977). *Tetramoriumtanakai* was resurrected from the synonymy with *T.kraepelini*; the worker characteristics mentioned by Bolton (1977), i.e. bicoloured body with very dark head and gaster and the long petiolar dorsum in *T.tanakai*, may be important, but in the queen caste, the configuration of the ocelli (very widely separated from each other) is quite distinctive amongst the *T.kraepelini*-complex. We have only two queens for *T.tanakai* (and one for the Japanese T.cf.kraepelini) so that our conclusion might remain tentative. However, our view would inspire further research on this difficult group over the entire Asian Region. Ocellar configuration can be a useful character for other *Tetramorium* species groups in the future when more material is amassed.

As mentioned earlier, there are many other useful queen characters. Some of them overlap with those traditionally used for workers. For example, the pilosity type on the antennal scape and mid- and hind-tibiae is generally common to both the worker and queen castes. On the other hand, the sculpture on the mesonotum in the queen is quite different from that of the worker. In most Japanese species the reticulation in the worker is replaced with longitudinal striae/rugae in the queen. Only in *T.lanuginosum* queens the rugae are irregular, mixed with reticulae. Rugose mesonotum in the worker caste is seen only in *T.smithi*. It is unknown which is more ancestral for *Tetramorium*, reticulation or striation/rugosity. Another difference between the castes is seen in the sculpture type of the mesopleuron; in the queen it tends to be more feebly sculptured than in the worker or nearly smooth in some species.

In conclusion, we think all the Japanese species can be properly identified also in the queen caste. The difficulty is getting males of all species because we have rarely found males in colonies in the field. However, captive colonies can produce winged sexuals through laboratory rearing for a long period. Cooperation between taxonomists and behavioural ecologists is indispensable for obtaining a complete set of material for the taxonomic study of winged ants.

## Supplementary Material

XML Treatment for Tetramorium bicarinatum

XML Treatment for Tetramorium cf. kraepelini

XML Treatment for Tetramorium lanuginosum

XML Treatment for Tetramorium nipponense

XML Treatment for Tetramorium pacificum

XML Treatment for Tetramorium simillimum

XML Treatment for Tetramorium smithi

XML Treatment for Tetramorium tanakai

XML Treatment for Tetramorium tonganum

XML Treatment for Tetramorium tsushimae

## References

[B1] Agavekar G Hita Garcia F Economo EP (2017) Taxonomic overview of the hyperdiverse and genus *Tetramorium* Mayr (Hymenopter, Formicidae) in India with descriptions and X-ray microtomography of two new species from the Andaman Islands. PeerJ 2017, 5: e3800. 10.7717/peerj.3800 PMC5610556 28948101

[B2] AntWiki (2021) *Tetramorium*. [online]. Available from antwiki org. [accessed 10 May 2021]

[B3] Bharti H Kumar R (2012) Taxonomic studies on genus *Tetramorium* Mayr (Hymenoptera, Formicidae) with report of two new species and three new records including a tramp species from India with a revised key. ZooKeys 207: 11–35. 10.3897/zookeys.207.3040 PMC3409682 22855638

[B4] Bolton B (1976) The ant tribe Tetramoriini (Hymenoptera: Formicidae). Constituent genera, review of smaller genera and revision of *Triglyphothrix* Forel. Bulletin of the British Museum (Natural History) Entomology Series 34: 283–379.

[B5] Bolton B (1977) The ant tribe Tetramoriini (Hymenoptera: Formicidae). The genus *Tetramorium* Mayr in the Oriental and Indo-Australian regions, and in Australia. Bulletin of the British Museum (Natural History) Entomology Series 36: 67–151.

[B6] Bolton B (1980) The ant tribe Tetramoriini (Hymenoptera: Formicidae). The genus *Tetramorium* Mayr in the Ethiopian zoogeographical region. Bulletin of the British Museum (Natural History) Entomology Series 40: 193–384.

[B7] Bolton B (1995) A New General Catalogue of the Ants of the World. Harvard University Press, Cambridge, Massachusetts & London, England, 512 pp.

[B8] Hita Garcia F Fisher B (2012) The ant genus *Tetramorium* Mayr (Hymenoptera: Formicidae) in the Malagasy region-taxonomy of the *T.bessonii*, *T.bonibony*, *T.dysalum*, *T.marginatum*, *T.tsingy*, and *T.weitzeckeri* species groups. Zootaxa 3365: 1–133. 10.11646/zootaxa.3365.1.1

[B9] Japanese Ant Database Group (2003) Ants of Japan. Gakken, Tokyo, e136. [In Japanese]

[B10] Ogata K (1991) A generic synopsis of the poneroid complex of the family Formicidae (Hymenoptera), Part. II. Subfamily Myrmicinae. Bulletin of the Institute of Tropical Agriculture, Kyushu University 14: 61–149.

[B11] Onoyama K (1980) An introduction to the ant fauna of Japan, with a check list (Hymenoptera, Formicidae). Kontyû, Tokyo 48: 193–212.

[B12] Santschi F (1928) Insects of Samoa and Other Samoan Territorial Archipelagos 5. Hymenoptera, Formicidae: 41–58.

[B13] Steiner FM Schlick-Steiner BC Moder K (2006) Morphology-based cyber identification engine to identify ants of the *Tetramoriumcaespitum*/*impurum* complex (Hymenoptera: Formicidae). Myrmecologische Nachrichten 8: 175–180.

[B14] Terayama M (2020) Formicidae. In: Editorial Committee of Catalogue of the Insects of Japan (Ed.) Catalogue of the Insects of Japan. Hymenoptera Vol. 9, Part 3: 85–160 Apocrita, Aculeata. Entomological Society of Japan. [Distributed by Touka Shobo, Fukuoka]

[B15] Terayama M Kubota S Eguchi K (2014) Encyclopedia of Japanese Ants. Asakura-shoten, Tokyo, 278 pp. [In Japanese]

[B16] Yamane Sk Fukumoto S (2017) Chapter 6. Tramp ant species in the Satsunan Islands, 108–131. In: Kagoshima University Biodiversity Study Group (Ed.) Alien Animals and Plants in the Amami Islands. Nanpô-shinsha, Kagoshima. [In Japanese]

[B17] Yamane Sk Hosoishi S (2020) Identification of the Japanese species of the ant genus *Pheidole* based on queen characters. Japanese Journal of Entomology (New Series) 23: 37–53. [In Japanese with English abstract and keys to species]

[B18] Yoshimura M (2020) Chapter 4. Taxonomy of alien ants, 34–50. In: Hashimoto Y. (Ed.) Gairaiari no Hanashi. Asakura Shoten, Tokyo. [In Japanese]

